# Timing of renal replacement therapy and long-term risk of chronic kidney disease and death in intensive care patients with acute kidney injury

**DOI:** 10.1186/s13054-017-1903-y

**Published:** 2017-12-28

**Authors:** Søren Christiansen, Steffen Christensen, Lars Pedersen, Henrik Gammelager, J. Bradley Layton, M. Alan Brookhart, Christian Fynbo Christiansen

**Affiliations:** 1 Department of Clinical Epidemiology, Aarhus University Hospital, Olof Palmes Allé 43-45, 8200 Aarhus N, Denmark; 2 Department of Anesthesiology and Intensive Care Medicine, Aarhus University Hospital, Brendstrupgaardsvej 100, 8200 Aarhus N, Denmark; 3 Department of Anesthesiology and Intensive Care Medicine, Viborg Regional Hospital, Heibergs Alle 4, 8800 Viborg, Denmark; 4 Department of Epidemiology, Gillings School of Global Public Health, University of North Carolina, Chapel Hill, NC USA

**Keywords:** Acute kidney injury, Chronic kidney disease, End-stage renal disease, Renal replacement therapy, Timing

## Abstract

**Background:**

The optimal time to initiate renal replacement therapy (RRT) in intensive care unit (ICU) patients with acute kidney injury (AKI) is unclear. We examined the impact of early RRT on long-term mortality, risk of chronic kidney disease (CKD), and end-stage renal disease (ESRD).

**Methods:**

This cohort study included all adult patients treated with continuous RRT in the ICU at Aarhus University Hospital, Skejby, Denmark (2005–2015). Data were obtained from a clinical information system and population-based registries. Early treatment was defined as RRT initiation at AKI stage 2 or below, and late treatment was defined as RRT initiation at AKI stage 3. Inverse probability of treatment (IPT) weights were computed from propensity scores. The IPT-weighted cumulative risk of CKD (estimated glomerular filtration rate < 60 ml/minute/1.73 m^2^), ESRD, and mortality was estimated and compared using IPT-weighted Cox regression.

**Results:**

The mortality, CKD, and ESRD analyses included 1213, 303, and 617 patients, respectively. The 90-day mortality in the early RRT group was 53.6% compared with 46.0% in the late RRT group (HR 1.24, 95% CI 1.03–1.48). The 90-day to 5-year mortality was 37.7% and 41.5% in the early and late RRT groups, respectively (HR 0.95, 95% CI 0.70–1.29). The 5-year risk of CKD was 35.9% in the early RRT group and 44.9% in the late RRT group (HR 0.74, 95% CI 0.46–1.18). The 5-year risk of ESRD was 13.3% in the early RRT group and 16.7% in the late RRT group (HR 0.79, 95% CI 0.47–1.32).

**Conclusions:**

Early initiation was associated with increased 90-day mortality. In patients surviving to day 90, early initiation was not associated with a major impact on long-term mortality or risk of CKD and ESRD. Despite potential residual confounding due to the observational design, our findings do not support that early RRT initiation is superior to late initiation.

**Electronic supplementary material:**

The online version of this article (doi:10.1186/s13054-017-1903-y) contains supplementary material, which is available to authorized users.

## Background

Acute kidney injury (AKI) occurs in 39–57% of intensive care unit (ICU) patients, and 6–14% of ICU patients are treated with renal replacement therapy (RRT) [1–4]. Patients with RRT-treated AKI have a 90-day mortality of 50–60% and a 5-year risk of end-stage renal disease (ESRD) of > 10% [5–8]. The optimal time to initiate RRT remains unclear [9]. There is a theoretical rationale for early initiation of RRT, such as improved control of fluid balance, electrolytes, and acid-base status. However, a treatment strategy of earlier initiation is accompanied by the risk of exposing patients who might have recovered without RRT to RRT and treatment-related complications [10].

Authors of meta-analyses of primarily observational studies have found early RRT initiation to be associated with reduced short-term mortality compared with late RRT initiation [11, 12]. However, the majority of included studies had methodological limitations, such as small sample sizes or inadequate control of confounding. Results of randomized controlled trials (RCTs) are conflicting, but authors of meta-analyses of pooled data have observed no difference in short-term mortality or RRT dependency [13, 14].

To our knowledge, only two observational studies have assessed mortality beyond day 90, and none have examined the impact of timing of RRT on risk of chronic kidney disease (CKD) and ESRD [15, 16]. To address this gap in the literature, we conducted a cohort study to examine the impact of early RRT in ICU patients on 5-year risk of CKD, ESRD, and mortality.

## Methods

### Study population and setting

This cohort study included all patients aged 15 years or older who were treated with continuous RRT in the 13-bed ICU at Aarhus University Hospital, Skejby, Denmark, from January 1^st^, 2005, to January 1^st^, 2015. Patients were admitted from departments of infectious diseases, cardiology, nephrology, urology, gynecology, thoracic surgery, and vascular surgery. The ICU is a university-affiliated, highly specialized referral unit treating cardiac transplant patients, among others, and performing extracorporeal membrane oxygenation (ECMO). Patients treated with RRT were identified in a clinical information system (CIS) database used in the ICU (Picis; Picis Inc., Wakefield, MA, USA). The CIS database contains detailed, real-time, electronically registered data on, among others, treatment with vasopressors and inotropes, mechanical ventilation, hemodynamics, and RRT treatment. Additionally, it contains manually registered data on weight and urine output. All patients were treated using a PRISMA continuous RRT system (GAMBRO DASCO S.p.A., Medolla, Italy) with a prescribed effluent dose of 30 ml/kg/h. Continuous venovenous hemodiafiltration was used in the vast majority (>90%) of patients. Until March 2014, heparin was the standard anticoagulation method, and citrate was used thereafter.

The Danish health care system is tax-funded and provides free and universal health care for all Danish citizens. Every citizen has a Danish civil registration (CPR) number assigned at birth or immigration [17]. Through the CPR number, deterministic individual-level linkage between clinical and administrative databases is possible. To ensure information on preadmission morbidity and follow-up, we included only patients with a Danish CPR number and who had residency in Denmark [17]. We considered only patients with newly developed severe renal impairment and therefore excluded patients with existing ESRD at ICU admission.

### Timing of renal replacement therapy

The AKI stage was assessed by using the Kidney Disease: Improving Global Outcomes (KDIGO) AKI criteria for plasma creatinine and urine output (measured as volume in ml/minute/kg; whichever value gave patients the highest stage) (Table 1) [18]. The creatinine measurements were obtained from a laboratory database [19]. The database contains information on every blood test taken during in- and outpatient visits to public or private hospitals or submitted by general practitioners in the central and northern regions of Denmark (for NPU [Nomenclature for Properties and Units] codes, *see* Additional file 1: Table S1). The creatinine ratio was calculated using the latest creatinine measurement 24 h before RRT initiation divided by the baseline creatinine. Baseline creatinine was estimated as the mean of all creatinine measurements from outpatient clinics or visits to the general practitioner from 1 year to 7 days before ICU admission [20]. For patients without outpatient creatinine measurements, the baseline was imputed with the four-variable Modification of Diet in Renal Disease (MDRD) equation (assuming an estimated glomerular filtration rate [eGFR] of 75 ml/minute/1.73 m^2^ and Caucasian race), as suggested by the KDIGO AKI guideline [18].

**Table 1:** Definition of early and late renal replacement therapy

Group	KDIGO stage	Creatinine	Urine output
Early RRT		Patients not meeting AKI criteria	
	1	1.5–1.9 times baseline or ≥ 26.5 μmol/L (0.3 mg/dl) increase in creatinine within 48 h	<0.5 ml/kg/h for 6–12 h
	2	2.0–2.9 times baseline	<0.5 ml/kg/h for > 12 h
Late RRT	3	3.0 times baseline or creatinine ≥ 354 μmol/L (4.0 mg/dl)^a^	<0.3 ml/kg/h for > 24 h or anuria for ≥ 12 h

*Abbreviations*: *AKI* Acute kidney injury, *KDIGO* Kidney Disease: Improving Global Outcomes, *RRT* Renal replacement therapy

^a^Satisfies AKI criteria as well

The urine output was obtained from the CIS, requiring at least 6 h of observation time in accordance with the KDIGO AKI criteria (Table 1) [18]. To compute urine output per kilogram of body weight, we used the average weight during the ICU stay. Patients who could not be evaluated for their urine output owing to < 6 h of observation time, no diuresis measurements in the specified time period before RRT, or no recorded weight, were assigned an AKI stage based on the creatinine measurements only.

Timing of RRT was defined as early if treatment was initiated at AKI stage 2 or below, including patients not meeting the AKI criteria. Late initiation of RRT was defined as AKI stage 3 at the time of RRT initiation.

### Chronic kidney disease, end-stage renal disease, and mortality

CKD was defined as at least two eGFR measurements < 60 ml/minute/1.73 m^2^ separated by > 90 days [21]. The eGFR was estimated from creatinine measurements using the four-variable MDRD equation (assuming Caucasian race) using only outpatient blood samples to avoid inclusion of creatinine measurements performed during hospitalization with acute illness.

ESRD was defined as treatment with chronic RRT or kidney transplant > 90 days after initiation of RRT in the ICU and was obtained from the Danish National Patient Registry (DNPR). The DNPR contains information on all nonpsychiatric admissions since 1977 and outpatient and emergency visits since 1995. The data include the type of admission (elective or acute), procedures performed, and up to 20 diagnoses given by the physician at discharge, using the Tenth Revision of the International Classification of Diseases since 1994 (Additional file 1: Table S1) [22]. With CKD and ESRD as outcomes of interest, we included both patients who had renal recovery and later developed the outcome and patients without renal recovery.

Time of death was obtained from the Danish Civil Registration system. The registry is updated daily and contains complete information since 1968 on vital status, emigration, and residency [17].

### Covariates

Information on preadmission morbidity 10 years before ICU admission was obtained from the DNPR and included diagnoses assigned after an in- or outpatient visit, except visits to the emergency department to increase validity [22]. We thus included previous diagnoses of renal disease, diabetes (types 1 and 2), congestive heart failure, myocardial infarction, cerebrovascular disease, chronic pulmonary disease, liver disease, periphery vascular disease, malignant solid tumors, lymphoma, leukemia, and metastatic solid tumors, all of which are considered accurate [22]. Using the DNPR, patients were categorized as nonsurgical, elective noncardiac surgical, acute noncardiac surgical, elective cardiac surgical, and acute cardiac surgical, dependent on their type of admission and surgery performed within a period from 24 h before ICU admission until RRT initiation [23]. Information on mechanical ventilation was obtained from the DNPR. Using information from the laboratory database and the CIS, we computed the patients’ Sequential Organ Failure Assessment (SOFA) score on the basis of worst values 24 h before initiation of RRT, not considering the Glasgow Coma Scale score and renal function (i.e., creatinine level) [24]. Missing or not measured physiological parameters included in the SOFA score were considered normal. The latest potassium and sodium measurements within 24 h before RRT initiation were obtained from the laboratory database [19].

### Statistical analysis

The patient’s characteristics, including demography, other ICU treatments, laboratory values, and time of treatment, were tabulated for the early and late initiation groups. Continuous variables were presented as means with standard deviations (SD) or medians with interquartile intervals (IQI), as appropriate. Categorical variables were presented as frequencies and percentages. We followed patients until outcome of interest, emigration, 5 years from RRT initiation, or censoring on January 1^st^, 2016, whichever came first. Owing to differences in follow-up requirements and outcome definitions, we performed an analysis for each outcome: CKD, ESRD, and death. By definition, chronic renal impairment should last > 90 days [21]. Therefore, we included only patients who survived until day 90 when we examined the impact on CKD and ESRD. Furthermore, to examine the impact of early and late RRT on the risk of CKD, we included only patients having residency in a Danish region covered by the laboratory database (i.e., the central and northern regions of Denmark) and without evidence of existing renal disease. Prior renal disease was defined as at least two outpatient eGFR measurements < 60 ml/minute/1.73 m^2^ at least 90 days apart during the year before ICU admission or a diagnosis of any renal disease before admission [19].

To adjust for potential confounders, propensity scores were estimated using a multivariable logistic regression model including all the prespecified characteristics presented for each cohort [25]. The included variables are presented in Table 2 for the mortality analysis, with the CKD and ESRD analyses presented in Additional file 2: Table S2 and Additional file 3: Table S3, respectively. Continuous variables were included in the model with a restricted cubic spline function with four knots. From the propensity scores, we computed inverse probability of treatment (IPT) weights; when these weights are applied to a population, they create a pseudo-population in which exposure status is independent of measured covariates [26, 27]. The covariate balance in the full and IPT-weighted cohorts was evaluated with standard mean differences [26]. For each outcome, we estimated the IPT-weighted cumulative risk. With ESRD and CKD as outcomes of interest, we accounted for death as a competing risk. The HRs were estimated using a crude and IPT-weighted Cox proportional hazards regression with the 95% CI estimated using a robust variance estimator. The assumption of proportional hazards was checked graphically by log(-log) plots. As sensitivity analyses, we repeated the analyses for each outcome after exclusion of patients treated with ECMO in addition to RRT during ICU admission. Additionally, we performed a fifth-percentile asymmetrical trim to remove the potential influence of unmeasured confounding in the tails of the propensity score distributions [28].

**Table 2:** Characteristics for the full and inverse probability of treatment-weighted cohort with mortality as outcome

	Full cohort			IPT-weighted cohort		
	Early RRT (*n* = 621)	Late RRT (*n* = 592)	SMD	Early RRT (*n* = 621)	Late RRT (*n* = 592)	SMD
Demographics						
Age, years, median (IQI)	67.7 (58.5–75.3)	69.0 (59.3–75.8)	−0.09	68.1 (59.5–76.6)	68.7 (58.1–75.5)	0.03
Male sex, *n* (%)	419 (65.5)	419 (70.8)	−0.11	425 (68.5)	401 (67.7)	0.02
Surgical status, *n* (%)						
Nonsurgical	229 (36.9)	281 (47.5)	−0.22	261 (42.1)	248 (42.0)	−0.02
Noncardiac surgery, elective	37 (6.0)	47 (7.9)	−0.08	42 (6.7)	43 (7.3)	−0.02
Noncardiac surgery, acute	67 (10.8)	66 (11.1)	−0.01	67 (10.8)	65 (11.0)	−0.00
Cardiac surgery, elective	100 (16.1)	66 (11.1)	0.14	84 (13.5)	74 (12.5)	0.03
Cardiac surgery, acute	188 (30.3)	132 (22.3)	0.18	167 (26.8)	161 (27.3)	−0.01
SOFA score, mean (SD)	5.5 (2.4)	4.8 (2.5)	0.29	5.2 (2.5)	5.2 (2.5)	0.01
ICU treatments, *n* (%)						
Vasopressors or inotropes	555 (89.4)	491 (82.9)	0.19	543 (87.4)	513 (86.6)	0.02
Mechanical ventilation	475 (76.5)	420 (70.9)	0.13	460 (74.0)	441 (74.5)	−0.01
Extracorporeal membrane oxygenation	84 (13.5)	40 (6.8)	0.23	67 (10.7)	65 (11.0)	−0.01
Laboratory values						
Creatinine, baseline, μmol/L, median (IQI)	94.9 (82.0–117.0)	90.7 (73.7–102.2)	0.19	93.2 (76.8–115.0)	92.0 (76.8–111.7)	0.01
Potassium, mmol/L, median (IQI)	4.4 (3.9–5.0)	4.5 (4.1–5.1)	−0.17	4.4 (4.0–5.0)	4.5 (4.0–5.0)	−0.04
Sodium, mmol/L, mean (SD)	139.3 (7.0)	138.8 (7.2)	0.06	138.9 (7.1)	138.8 (7.0)	0.00
Preadmission morbidity, *n* (%)						
Renal disease	178 (28.7)	208 (35.1)	−0.14	198 (31.9)	188 (31.8)	0.00
Diabetes	101 (16.3)	107 (18.1)	−0.05	108 (17.4)	104 (17.5)	−0.00
Congestive heart disease	180 (29.0)	120 (20.3)	0.20	161 (25.9)	153 (25.9)	−0.00
Myocardial infarction	154 (24.8)	136 (23.0)	0.04	149 (24.0)	148 (25.0)	−0.02
Cerebrovascular disease	80 (12.9)	86 (14.5)	−0.05	83 (13.3)	76 (12.9)	0.01
Chronic pulmonary disease	113 (18.2)	90 (15.2)	0.08	101 (16.3)	92 (15.6)	0.02
Liver disease	21 (3.4)	23 (3.9)	−0.03	22 (3.6)	21 (3.5)	0.00
Vascular disease	172 (27.7)	161 (27.2)	0.01	180 (29.1)	164 (27.7)	0.03
Tumor	68 (11.0)	95 (16.0)	−0.15	85 (13.7)	81 (13.7)	−0.00
Lymphoma	9 (1.4)	6 (1.0)	0.04	8 (1.3)	9 (1.5)	−0.01
Leukemia	7 (1.1)	5 (0.8)	0.03	6 (0.9)	7 (1.1)	−0.02
Metastasis	16 (2.6)	17 (2.9)	−0.02	16 (2.5)	17 (2.9)	−0.03
Year of treatment, *n* (%)						
2005–2006	95 (15.3)	149 (25.2)	−0.25	121 (19.5)	115 (19.4)	−0.04
2007–2008	101 (16.3)	120 (20.3)	−0.10	112 (18.0)	106 (18.0)	−0.00
2009–2010	141 (22.7)	93 (15.7)	0.18	119 (19.1)	119 (20.1)	−0.03
2011–2012	158 (25.4)	94 (15.9)	0.24	132 (21.3)	124 (20.9)	0.01
2013–2014	126 (20.3)	136 (23.0)	−0.07	138 (22.1)	128 (21.7)	0.01

*Abbreviations*: *ICU* Intensive care unit, *IPT* Inverse probability of treatment, *IQI* Interquartile interval, *RRT* Renal replacement therapy, *SMD* Standard mean difference, *SOFA* Sequential Organ Failure Assessment, *SD* Standard deviation

Analyses were performed using the Stata version 14.1 statistical software package (StataCorp LP, College Station, TX, USA). The study was approved by the Danish Data Protection Agency (record number 2015-57-0002, Aarhus University record number 2016-051-000001-432).

## Results

### Descriptive results

**Fig. 1:**
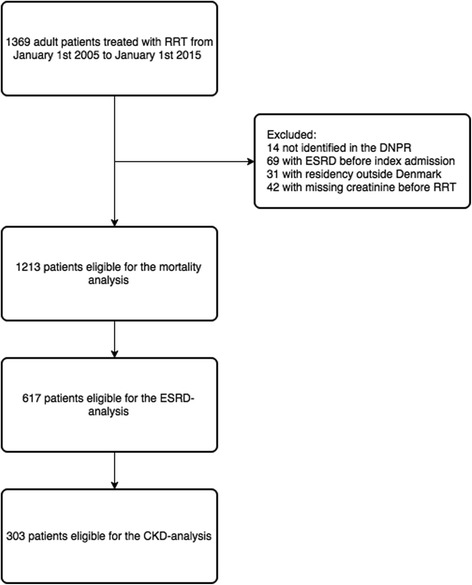
Flowchart of eligible patients included in the analyses. *CKD* Chronic kidney disease, *DNPR* Danish National Patient Registry, *ESRD* End-stage renal disease, *RRT* Renal replacement therapy

In the 10-year study period, 1369 adult patients were treated with RRT. We excluded 69 patients with previous ESRD, 31 with residency outside Denmark, and 14 who could not be linked to the DNPR. We had to exclude 42 patients because of missing information on laboratory values prior to RRT initiation. This gave us a study population of 1213 patients (Fig. 1). The total follow-up time was 1994 person-years. The median time from ICU admission to RRT initiation was 18.9 (IQI 8.3–35.2) h in the early group compared with 32.8 (IQI 7.8–79.8) h in the late group.

Characteristics of the full and IPT-weighted cohort with mortality as an outcome are presented in Table 2. In the full cohort, early and late RRT were initiated in 621 (51.2%) and 592 (48.8%) patients, respectively. The groups had similar age and sex distributions; however, early initiators had a higher SOFA score (5.5 vs. 4.8) and were more likely to have received acute cardiac surgery (30.3% vs. 22.3%). A higher proportion of patients in the late group were treated in the beginning of the study period (2005–2006). In the IPT-weighted cohort, demographics, use of other organ-supportive treatments, laboratory values, preadmission morbidities, and year of treatment were similarly distributed in both groups. The most frequent preadmission morbidities were renal disease, congestive heart failure, and vascular disease.

Measured baseline creatinine was available in 829 (68.3%) patients. Patients without measured baseline creatinine were equally distributed between both treatment groups, and they were younger and had less preadmission morbidity than those with baseline creatinine (Additional file 4: Table S4). AKI stage was assessed by urine output and creatinine criteria in 682 (56.2%) patients and by creatinine criteria only in 531 (43.8%) patients.

### Mortality

**Fig. 2:**
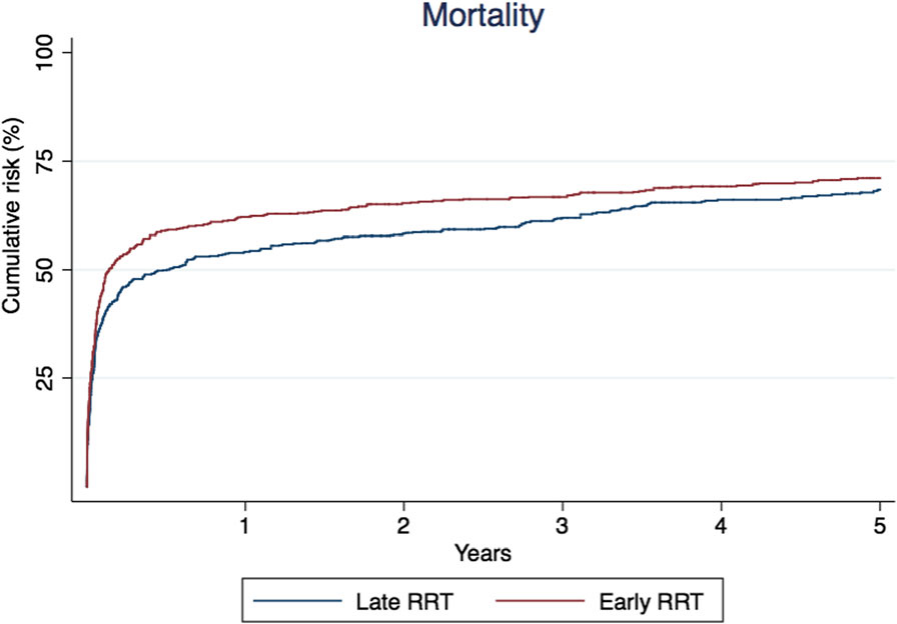
Inverse probability of treatment-weighted cumulative mortality for 0 to 90 days, HR 1.24 (95% CI 1.03–1.48); for 90 days to 5 years, HR 0.95 (95% CI 0.70–1.29). *RRT* Renal replacement therapy

In the crude analysis, 90-day mortality was 52.5% and 45.6% in the early and late groups, respectively (Table 3). The corresponding HR was estimated as 1.24 (95% CI 1.06–1.46) in the early group compared with the late group. In the IPT-weighted analysis, the 90-day mortality was 53.6% in the early group and 46.0% in late group (Table 3 and Fig. 2). This corresponds to a HR of 1.24 (95% CI 1.03–1.48). In the crude analysis, 90-day to 5-year mortality was 36.1% in the early group and 42.6% in the late group. The corresponding HR was estimated to 0.89 (95% CI 0.68–1.17). In the IPT-weighted analysis, the 90-day to 5-year mortality was 37.7% in the early group and 41.5% in the late group with a corresponding HR of 0.95 (95% CI 0.70–1.29).

**Table 3:** Crude and inverse probability of treatment-weighted cumulative risks and hazard ratios

	Crude analysis			IPT-weighted analysis		
	Early RRT, %	Late RRT, %	HR (95% CI)	Early RRT, %	Late RRT, %	HR (95% CI)
Mortality, *n* = 1213						
0 to 90 days	52.5	45.6	1.24 (1.06–1.46)	53.6	46.0	1.24 (1.03–1.48)
90 days to 5 years	36.1	42.6	0.89 (0.68–1.17)	37.7	41.5	0.95 (0.70–1.29)
0 to 5 years	69.7	68.8	NA	71.1	68.4	NA
CKD, *n* = 303						
90 days to 5 years	43.6	45.0	0.95 (0.67–1.34)	35.9	44.9	0.74 (0.46–1.18)
ESRD, *n* = 617						
90 days to 5 years	13.9	14.4	0.96 (0.62–1.48)	13.3	16.7	0.79 (0.47–1.32)

*Abbreviations*: *CI *Confidence interval, *CKD* Chronic kidney disease, *ESRD* End-stage renal disease, *NA* Not applicable, *IPT* Inverse probability of treatment, *RRT* Renal replacement therapy

### Chronic kidney disease

**Fig. 3:**
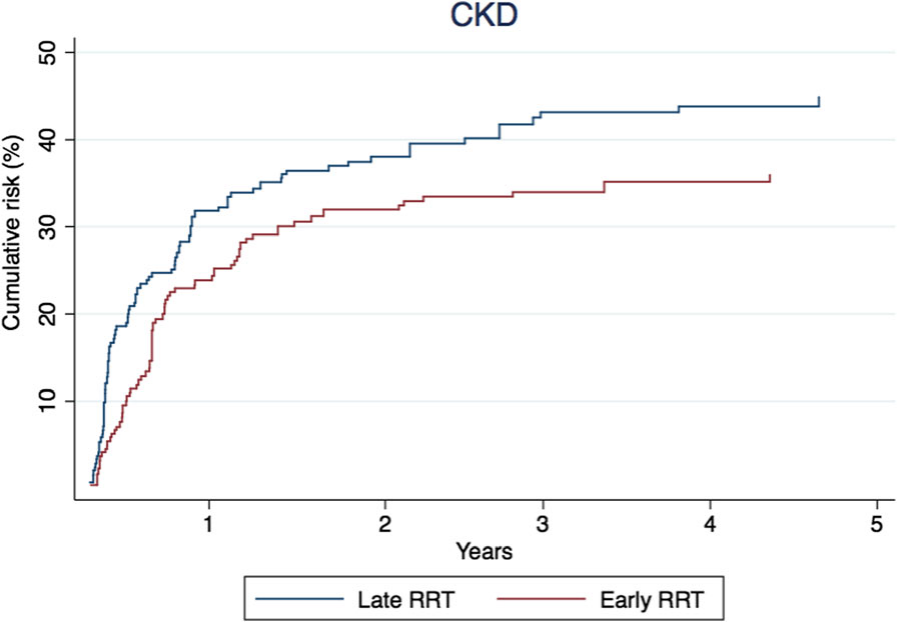
Inverse probability of treatment-weighted cumulative risk of chronic kidney disease for 90 days to 5 years, HR 0.74 (95% CI 0.46–1.18). *CKD* Chronic kidney disease, *RRT* Renal replacement therapy

With CKD as outcome, 303 patients were eligible for the analysis (Fig. 1). The patients’ characteristics were equally distributed between the early and late groups after IPT weighting (Additional file 2: Table S2). Of the 141 patients who initiated RRT early, 119 (84.4%) had 2 or more outpatient creatinine measurements after hospital discharge compared with 146 (90.1%) of the 162 patients who initiated RRT late. In the crude analysis, the 5-year risk of CKD was 43.6% and 45.0% in the early and late groups, respectively (Table 3). This corresponds to a HR of 0.95 (95% CI 0.67–1.34). In the IPT-weighted analysis, the 5-year risk of CKD was 35.9% in the early group and 44.9% in the late group with a corresponding HR of 0.74 (95% CI 0.46–1.18) (Table 3 and Fig. 3).

### End-stage renal disease

**Fig. 4:**
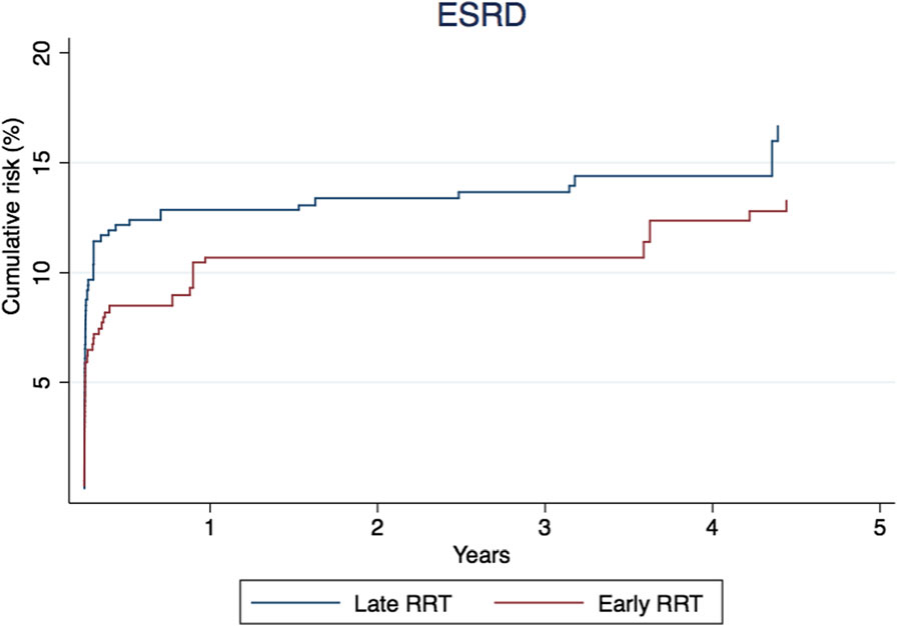
Inverse probability of treatment-weighted cumulative risk of end-stage renal disease for 90 days to 5 years, HR 0.79 (95% CI 0.47–1.32). *ESRD* End-stage renal disease, *RRT* Renal replacement therapy

With ESRD as outcome, the analysis included 617 patients (Fig. 1). After IPT weighting, the patients’ characteristics were equally distributed between the early and late groups (Additional file 3: Table S3). In the crude analysis, the 5-year risk of ESRD was 13.9% and 14.4% in the early and late groups, respectively (Table 3). This corresponds to a HR of 0.96 (95% CI 0.62–1.48). In the IPT-weighted analysis, the 5-year risk of ESRD was 13.3% in the early group and 16.7% in the late group (Table 3 and Fig. 4). This corresponds to a HR of 0.79 (95% CI 0.47–1.32).

### Sensitivity analyses

After exclusion of 149 patients treated with ECMO during ICU admission, the results were similar (Additional file 5: Table S5). Furthermore, after a fifth percentile asymmetrical trimming, the results of the analyses with mortality and CKD as outcomes of interest were similar (Additional file 5: Table S5), although for the ESRD outcome, the HR was attenuated from 0.79 (95% CI 0.47–1.32) to 1.02 (95% CI 0.59–1.75), indicating some treatment heterogeneity and possible residual confounding.

## Discussion

Using data collected from high-quality clinical and administrative registries, we found no clear evidence that early initiation of RRT in ICU patients was associated with improved long-term clinical outcomes. Early initiation of RRT was not associated with decreased long-term risk of CKD or ESRD. Early initiation was associated with increased short-term mortality but not with long-term mortality. Despite the use of advanced methods, we cannot rule out the influence of residual confounding.

### Existing studies

Until recently, existing studies on timing of RRT in ICU patients with AKI were mainly observational, with few RCTs conducted. Researchers in observational studies have used a wide array of parameters to define timing, such as blood urea nitrogen, creatinine, urine output, fluid overload, traditional indications for RRT (e.g., hyperkalemia), time from ICU admission, time from AKI diagnosis, and according to the RIFLE (risk, injury, failure, loss, end-stage renal disease) criteria [11]. The results derived from these observational studies on short-term mortality are conflicting, but after pooling the results of both observational and RCTs, meta-analyses in general favor early RRT initiation [11, 12]. However, because of the heterogeneity in definitions of early and late RRT and the methodological limitations of included studies, the results should be interpreted with caution. Wierstra et al. found that low-quality studies (mainly observational studies) favored early initiation (OR 0.47, 95% CI 0.34–0.65; early compared with late); however, in high-quality studies (mainly RCTs), early RRT did not statistically significantly reduce mortality (OR 0.67, 95% CI 0.38–1.15) [11].

In the majority of conducted RCTs, investigators have defined timing according to the presence of absolute indications [13]. In the recent multicenter AKIKI (Artificial Kidney Initiation in Kidney Injury) trial, 619 patients with AKI stage 3 who required mechanical ventilation, catecholamine infusion, or both were randomized either to immediate RRT (early) or to treatment being withheld until absolute indications developed (late) [29]. The authors observed no difference in 60-day mortality or RRT dependency. These findings are supported by meta-analyses including only RCTs on timing of RRT [13, 14]. The single-center ELAIN (Early versus Late Initiation of Renal-Replacement Therapy in Critically Ill Patients with Acute Kidney Injury) trial is the only RCT in which researchers have observed a lower short-term mortality with early initiation [30]. The authors randomized 231 patients with AKI stage 2 and plasma neutrophil gelatinase-associated lipocalin > 150 ng/ml to early RRT treatment (initiated within 8 h of diagnosis of AKI stage 2) or to late RRT treatment (within 12 h of AKI stage 3 diagnosis or if absolute indications developed). The authors found a reduced 90-day mortality with early initiation (HR 0.66, 95% CI 0.45–0.97); however, no difference in RRT dependency at day 90 was found.

No RCTs and few observational studies have assessed outcomes beyond day 90. Evaluating long-term outcomes is important because AKI is associated with a significant impact on long-term morbidity and mortality [8]. Researchers in two studies have examined the association between timing of RRT and mortality beyond day 90. Park et al. classified 607 patients as early or late initiators based on the median 6-h urine output [15]. Carl et al. defined 147 nonsurgical ICU patients as early or late initiators if their blood urea nitrogen was below or above 100 mg/dl (35 mmol/L), respectively [16]. In contrast to the present study, both of these previous studies demonstrated that early initiation was associated with reduced mortality; however, the interpretation of these studies was hampered by lack of control for important potential confounders.

### Strengths and limitations

Our study has some limitations that need to be considered when interpreting the results. First, we were able to include only patients who were treated with RRT. The lack of patients in the comparison group with AKI stage 2 (and thus potentially early initiators) who regained their renal function without RRT may have introduced bias. Second, baseline creatinine was available for 68.2% of the patients, with the remainder being estimated with the MDRD equation. This method may lead to slight over- and underestimation of the AKI stage [31–33]. Because the proportion of patients without available baseline creatinine were equally distributed between early and late RRT, any misclassification of AKI stage would most likely bias the estimates toward the null. Examination of patients with missing baseline creatinine showed that they were younger and had less comorbidity. Therefore, we found that imputation using the MDRD equation was reasonable. Third, we were able to obtain information on urine output in slightly more than half of the patients, with the remainder being staged only according to their creatinine ratio. This may have led to misclassification of the early and late initiation groups and may have biased the estimates. Fourth, because CKD can be present without symptoms, our outcome is dependent upon patients having an outpatient blood samples with creatinine taken. A large proportion of the patients included in the CKD analysis had two or more outpatient measurements, and we therefore find it plausible that we identified the vast majority of patients with CKD. Also, we have no reason to believe that AKI stage at initiation of RRT would impact health care vigilance after discharge, and therefore it cannot explain the observed results. Last, as with all observational studies, there still is a risk of residual confounding; however, we were able to adjust for demographic characteristics, detailed data on other ICU treatments, and laboratory values at initiation of RRT.

## Conclusions

Our findings do not support that early RRT initiation is superior to late initiation. Although early RRT was associated with increased short-term mortality, we found no association between timing and long-term outcomes. This study extends current knowledge on timing of RRT and short-term mortality by providing estimates of the impact on long-term mortality and risk of CKD and ESRD.

## Additional files

Additional file 1: Table S1. Codes used in the present study. (DOC 44 kb)

Additional file 2: Table S2. Characteristics for the full and IPT-weighted cohort with CKD as outcome. (DOC 67 kb)

Additional file 3: Table S3. Characteristics for the full and IPT-weighted cohort with ESRD as outcome. (DOC 78 kb)

Additional file 4: Table S4. Description of patients with and without baseline creatinine. (DOC 53 kb)

Additional file 5: Table S5. Sensitivity analyses. (DOC 41 kb)

## Abbreviations

AKI: Acute kidney injury
AKIKI: Artificial Kidney Initiation in Kidney Injury trial
CIS: Clinical information system
CKD: Chronic kidney disease
CPR: Danish civil registration number
DNPR: Danish National Patient Registry
ECMO: Extracorporeal membrane oxygenation
eGFR: Estimated glomerular filtration rate
ELAIN: Early versus Late Initiation of Renal-Replacement Therapy in Critically Ill Patients with Acute Kidney Injury trial
ESRD: End-stage renal disease
ICU: Intensive care unit
IPT: Inverse probability of treatment
IQI: Interquartile interval
KDIGO: Kidney Disease: Improving Global Outcomes
MDRD: Modification of Diet in Renal Disease
NPU: Nomenclature for Properties and Units
RCT: Randomized controlled trial
RIFLE: Risk, injury, failure, loss, end-stage renal disease criteria
RRT: Renal replacement therapy
SD: Standard deviations
SMD: Standard mean difference
SOFA: Sequential Organ Failure Assessment
