# Supplementary material for: Timing of renal replacement therapy and long-term risk of chronic kidney disease and death in intensive care patients with acute kidney injury
Source: Crit Care. 2017 Dec 28;21:326. doi: 10.1186/s13054-017-1903-y (PMC5745999; doi:10.1186/s13054-017-1903-y)
Supplement: Supplementary file 1 — Codes used in the present study. (DOC 44 kb) [file 13054_2017_1903_MOESM1_ESM.doc]

**Additional file 1: Table S1 C**odes used in current study

| **Description** | **Codes** |
| --- | --- |
| ***Preadmission morbidity (ICD-10)*** |  |
| Myocardial infarction | I21, I22, I23 |
| Congestive heart failure | I50, I11.0, I13.0, I13.2 |
| Peripheral vascular disease | I70, I71, I72, I73, I74, I77 |
| Chronic pulmonary disease | J40-J47, J60-J67, J68.4, J70.1, J70.3, J84.1, J92.0, J96.1, J98.2, J98.3 |
| Diabetes | E10.0, E10.1, E10.9, E11.0, E11.1, E11.9 |
| Renal disease | I12, I13, N00-N05, N07, N11, N14, N18-N19, Q61 |
| Any tumor | C00-C75 |
| Leukemia | C81-C85, C88, C90, C96 |
| Lymphoma | C81-C85, C88, C90, C96 |
| Metastatic solid tumor | C76-C80 |
| Liver disease | B15.0; B16.0; B16.2; B19.0; K70.4; K72; K76.6; I85  B18; K70.0-K70.3; K70.9; K71; K73; K74; K76.0 |
| ***Treatment (Nordic surgical codes and Danish procedure codes)*** |  |
| Non-cardiac surgery | KA, KB, KC, KD, KE, KG, KH, KJ, KK, KL, KM, KN, KP, KQ, KX, KY |
| Cardiac surgery | KF |
| Mechanical ventilation | BGDA0 |
| ***Outcomes (ESRD)*** |  |
| Chronic renal replacement therapy | BKFD2 |
| Kidney transplant | KKAS |
| ***Laboratory measurements (NPU codes and local analysis numbers)*** |  |
| Creatinine | NPU26918, NPU04998, NPU01807, NPU18016, NPU17559, NPU09101, NPU18105, ASS00354, ASS00355, ASS00356, 110266, 111016, 1311235, 1411235, 1511235, 1511236, 1511237, 1610154, 1610296, 1611807, 1710301, 1710552, 1711807, 1811807, 1817156, 1817428, 18016, 1155, 38927, 4998 |
| Potassium | NPU03230, ASS00102, ASS00255, 110262, 111262, 1311140, 1411140, 1511140, 1610147, 1613230, 1710304, 1713230, 1813230, 1817159 |
| Sodium | NPU03429, ASS00101, ASS00256, 110261, 1311170, 1411170, 1511170, 1610146, 1713429, 1813429 |
| Bilirubin | NPU01370, NPU01366, 110270, 1311218, 1411218 , 1511218 , 1610191, 1711370, 1811370, 110476, 1522032, 1722032, 1822032 |
| Platelets | NPU03568, NPU17586, AAA00946, 122576, 122587, 122676, 1313160, 1413160, 1523077, 1610113, 1813568, 1510899, 1710946 |
| Pa-O2 | NPU08977, NPU03009, NPU14104, 111029, 122063, 1324064, 1424064, 1622196, 1622286 , 1722184, 1722663, 1817282, 1817514, 1822184, 1524074 |
