# Supplementary material for: Timing of renal replacement therapy and long-term risk of chronic kidney disease and death in intensive care patients with acute kidney injury
Source: Crit Care. 2017 Dec 28;21:326. doi: 10.1186/s13054-017-1903-y (PMC5745999; doi:10.1186/s13054-017-1903-y)
Supplement: Supplementary file 2 — Characteristics for the full and IPT-weighted cohort with CKD as outcome. (DOC 67 kb) [file 13054_2017_1903_MOESM2_ESM.doc]

**Additional file 2: Table S2 Characteristics for the full and IPT-weighted cohort with CKD as outcome**.

|  | Full cohort | |  | IPT-Weighted cohort | |  |
| --- | --- | --- | --- | --- | --- | --- |
|  | Early RRT | Late RRT | SMD | Early RRT | Late RRT | SMD |
| N | 141 | 162 |  | 141 | 162 |  |
| **Demography** |  |  |  |  |  |  |
| Age, median (IQI) | 66.2 (56.4-73.2) | 66.8 (52.8-72.6) | 0.03 | 63.9 (56.3-72.5) | 66.8 (52.8-74.1) | -0.04 |
| Male, n (%) | 88 (62.4) | 122 (75.3) | -0.28 | 101 (71.7) | 114 (70.5) | 0.03 |
| Surgical status, n (%) |  |  |  |  |  |  |
| Non-surgical | 43 (30.5) | 59 (36.4) | -0.13 | 44 (31.3) | 54 (33.2) | -0.11 |
| Non-cardiac surgery, elective | 15 (10.6) | 15 (9.3) | 0.05 | 13 (9.3) | 17 (10.3) | -0.04 |
| Non-cardiac surgery, acute | 10 (7.1) | 27 (16.7) | -0.30 | 25 (18.0) | 20 (12.5) | 0.18 |
| Cardiac surgery, elective | 28 (19.9) | 21 (13.0) | 0.19 | 22 (15.6) | 28 (17.1) | -0.04 |
| Cardiac surgery, acute | 45 (31.9) | 40 (24.7) | 0.16 | 36 (25.8) | 44 (27.0) | -0.03 |
| SOFA score, mean (SD) | 6.0 (2.1) | 4.9 (2.6) | 0.46 | 5.3 (2.4) | 5.4 (2.4) | -0.03 |
| **ICU treatments, n (%)** |  |  |  |  |  |  |
| Vasopressors or inotropes | 135 (95.7) | 136 (84.0) | 0.40 | 124 (87.8) | 144 (89.1) | -0.04 |
| Mechanical ventilation | 115 (81.6) | 126 (77.8) | 0.09 | 116 (82.5) | 131 (80.8) | 0.04 |
| Extracorporeal membrane oxygenation | 21 (14.9) | 10 (6.2) | 0.29 | 14 (9.7) | 12 (7.4) | 0.08 |
| **Laboratory values** |  |  |  |  |  |  |
| Creatinine, baseline, µmol/L, median (IQI) | 90.3 (74.5-98.0) | 85.3 (73.5-96.0) | 0.17 | 90.2 (73.8-96.0) | 88.0 (74.6-97.3) | 0.00 |
| **Preadmission morbidities, n (%)** | |  |  |  |  |  |
| Diabetes | 14 (9.9) | 31 (19.1) | -0.26 | 17 (12.4) | 27 (16.7) | -0.12 |
| Cardiovascular disease^a^ | 79 (56.0) | 97 (59.9) | -0.08 | 91 (64.8) | 100 (61.9) | 0.06 |
| Neoplasm^b^ | 15 (10.6) | 29 (17.9) | -0.21 | 19 (13.8) | 24 (14.6) | -0.02 |
| **Year of treatment, n (%)** |  |  |  |  |  |  |
| 2005-2006 | 16 (11.3) | 29 (17.9) | -0.19 | 17 (12.3) | 23 (14.5) | 0.01 |
| 2007-2008 | 24 (17.0) | 25 (15.4) | 0.04 | 33 (23.2) | 30 (18.4) | 0.12 |
| 2009-2010 | 36 (25.5) | 24 (14.8) | 0.27 | 24 (17.0) | 28 (17.6) | -0.01 |
| 2011-2012 | 34 (24.1) | 31 (19.1) | 0.12 | 33 (23.3) | 36 (22.0) | 0.03 |
| 2013-2014 | 31 (22.0) | 53 (32.7) | -0.24 | 34 (24.2) | 45 (27.5) | -0.08 |
| ^a^ Myocardial infarction, congestive heart disease, cerebrovascular disease, vascular disease.  ^b^ Tumor, leukemia, lymphoma, metastasis.  Abbreviations: ICU: intensive care unit, IQI: interquartile interval, N: number, RRT: Renal replacement therapy, SD: Standard deviation, SMD: Standard mean difference, SOFA: sequential organ assessment score. | | | | | | |
|
|
