# Supplementary material for: Timing of renal replacement therapy and long-term risk of chronic kidney disease and death in intensive care patients with acute kidney injury
Source: Crit Care. 2017 Dec 28;21:326. doi: 10.1186/s13054-017-1903-y (PMC5745999; doi:10.1186/s13054-017-1903-y)
Supplement: Supplementary file 3 — Characteristics for the full and IPT-weighted cohort with ESRD as outcome. (DOC 78 kb) [file 13054_2017_1903_MOESM3_ESM.doc]

**Additional file 3: Table S3** Characteristics for the full and IPT-weighted cohort with ESRD as outcome.

|  | Full cohort | | | |  | | IPT-weighted cohort | | | |  |
| --- | --- | --- | --- | --- | --- | --- | --- | --- | --- | --- | --- |
|  | Early RRT | | Late RRT | | SMD | | Early RRT | | Late RRT | | SMD |
| N | 295 | | 322 | |  | | 295 | | 322 | |  |
| **Demography** |  | |  | |  | |  | |  | |  |
| Age, median (IQI) | 63.9 (53.4-72.5) | | 66.8 (55.8-73.4) | | -0.10 | | 64.9 (55.4-72.7) | | 64.8 (52.1-73.1) | | 0.09 |
| Male, n (%) | 231 (65.4) | | 231 (71.7) | | -0.14 | | 205 (69.5) | | 217 (67.3) | | 0.05 |
| Surgical status, n (%) |  | |  | |  | |  | |  | |  |
| Non-surgical | 99 (33.6) | | 151 (46.9) | | -0.27 | | 118 (40.0) | | 132 (41.1) | | 0.02 |
| Non-cardiac surgery, elective | 21 (7.1) | | 26 (8.1) | | -0.04 | | 22 (7.3) | | 26 (7.9) | | -0.02 |
| Non-cardiac surgery, acute | 21 (7.1) | | 37 (11.5) | | -0.15 | | 27 (9.3) | | 29 (8.9) | | 0.01 |
| Cardiac surgery, elective | 59 (20.0) | | 41 (12.7) | | 0.20 | | 47 (15.8) | | 47 (14.7) | | 0.03 |
| Cardiac surgery, acute | 95 (32.2) | | 67 (20.8) | | 0.26 | | 81 (27.5) | | 88 (27.4) | | 0.00 |
| SOFA score, mean | 5.5 (2.4) | | 4.6 (2.6) | | 0.38 | | 5.1 (2.5) | | 5.1 (2.5) | | 0.01 |
| **ICU treatments, n (%)** |  | |  | |  | |  | |  | |  |
| Vasopressors or inotropes | 263 (89.2) | | 254 (78.9) | | 0.28 | | 249 (84.6) | | 271 (84.1) | | 0.01 |
| Mechanical ventilation | 235 (79.7) | | 229 (71.1) | | 0.20 | | 230 (78.0) | | 246 (76.4) | | 0.04 |
| Extracorporeal membrane oxygenation | 47 (15.9) | | 23 (7.1) | | 0.28 | | 36 (12.3) | | 39 (12.0) | | 0.01 |
| **Laboratory values** |  | |  | |  | |  | |  | |  |
| Creatinine, baseline, µmol/L, median (IQI) | 95.0 (80.5-116.0) | | 91.4 (74.0-102.6) | | 0.19 | | 94.0 (78.7-115.0) | | 93.3 (79.0-111.7) | | -0.06 |
| Potassium, mmol/L, median (IQI) | 4.4 (4.0- 5.0) | | 4.5 (4.1- 5.0) | | -0.20 | | 4.5 (4.1-5.0) | | 4.5 (4.1-5.0) | | -0.08 |
| Sodium, mmol/L, mean (SD) | 139.2 (6.5) | | 138.1 (7.3) | | 0.16 | | 138.5 (6.7) | | 138.4 (6.9) | | 0.01 |
| **Preadmission morbidity, n (%)** | |  | |  | |  | |  | |  | |
| Renal disease | 86 (29.2) | | 122 (37.9) | | -0.19 | | 105 (35.5) | | 115 (35.6) | | -0.00 |
| Diabetes | 41 (13.9) | | 59 (18.3) | | -0.12 | | 47 (16.0) | | 55 (17.0) | | -0.03 |
| Congestive heart disease | 79 (26.8) | | 59 (18.3) | | 0.20 | | 69 (23.5) | | 75 (23.2) | | 0.01 |
| Myocardial infarction | 59 (20.0) | | 58 (18.0) | | 0.05 | | 57 (19.2) | | 63 (19.5) | | -0.01 |
| Cerebrovascular disease | 28 (9.5) | | 42 (13.0) | | -0.11 | | 33 (11.3) | | 34 (10.6) | | 0.02 |
| Chronic pulmonary disease | 40 (13.6) | | 41 (12.7) | | 0.02 | | 39 (13.3) | | 41 (12.7) | | 0.02 |
| Liver disease | 5 (1.7) | | 14 (4.3) | | -0.16 | | 6 (1.9) | | 9 (2.8) | | -0.05 |
| Vascular disease | 72 (24.4) | | 91 (28.3) | | -0.09 | | 86 (29.0) | | 86 (26.6) | | 0.06 |
| Neoplasm^a^ | 30 (10.2) | | 54 (16.8) | | -0.19 | | 40 (13.4) | | 45 (13.8) | | -0.01 |
| **Year of treatment, n (%)** |  | |  | |  | |  | |  | |  |
| 2005-2006 | 37 (12.5) | | 72 (22.4) | | -0.26 | | 49 (16.7) | | 53 (16.6) | | -0.03 |
| 2007-2008 | 43 (14.6) | | 58 (18.0) | | -0.09 | | 47 (15.8) | | 57 (17.8) | | -0.05 |
| 2009-2010 | 79 (26.8) | | 54 (16.8) | | 0.24 | | 66 (22.2) | | 73 (22.7) | | -0.01 |
| 2011-2012 | 76 (25.8) | | 55 (17.1) | | 0.21 | | 65 (22.0) | | 66 (20.5) | | 0.04 |
| 2013-2014 | 60 (20.3) | | 83 (25.8) | | -0.13 | | 69 (23.2) | | 72 (22.4) | | 0.02 |
| ^a^ Tumor, leukemia, lymphoma, metastasis.  Abbreviations: ICU: intensive care unit, IQI: interquartile interval, N: number, RRT: Renal replacement therapy, SD: Standard deviation, SMD: Standard mean difference, SOFA: sequential organ assessment score. | | | | | | | | | | | |
|
