# Supplementary material for: Timing of renal replacement therapy and long-term risk of chronic kidney disease and death in intensive care patients with acute kidney injury
Source: Crit Care. 2017 Dec 28;21:326. doi: 10.1186/s13054-017-1903-y (PMC5745999; doi:10.1186/s13054-017-1903-y)
Supplement: Supplementary file 4 — Description of patients with and without baseline creatinine. (DOC 53 kb) [file 13054_2017_1903_MOESM4_ESM.doc]

**Additional file 4: Table S4** Description of patients with and without baseline creatinine.

|  | Not missing  n (%) | Missing  n (%) |
| --- | --- | --- |
|  | 829 (68.3) | 384 (31.7) |
| Early RRT, n (%) | 423 (68.1) | 198 (31.9) |
| Late RRT, n (%) | 406 (68.6) | 186 (31.4) |
| **Demography** |  |  |
| Age, median (IQI) | 69.0 (59.3-75.8) | 67.7 (58.5-75.3) |
| Male, n (%) | 551 (70.8) | 275 (65.5) |
| **Surgical status, n (%)** |  |  |
| Non-surgical | 375 (45.2) | 135 (35.2) |
| Non-cardiac surgery, elective | 79 (9.5) | 5 (1.3) |
| Non-cardiac surgery, acute | 88 (10.6) | 45 (11.7) |
| Cardiac surgery, elective | 121 (14.6) | 45 (11.7) |
| Cardiac surgery, acute | 166 (20.0) | 154 (40.1) |
| **SOFA score** | 5.1 (2.4) | 5.4 (2.7) |
| **ICU treatments, n (%)** |  |  |
| Vasopressor or inotropes | 715 (86.2) | 331 (86.2) |
| Mechanical ventilation | 609 (73.5) | 286 (74.5) |
| Extracorporeal membrane oxygenation | 44 (5.3) | 80 (20.8) |
| **Laboratory values** |  |  |
| Potassium, mmol/L, median (IQI) | 4.5 (4.0- 5.0) | 4.4 (4.0- 5.0) |
| Sodium, mmol/L, mean (SD) | 138.7 (6.9) | 139.9 (7.5) |
| **Preadmission morbidity, n (%)** | |  |
| Renal disease | 285 (34.4) | 101 (26.3) |
| Diabetes | 179 (21.6) | 29 (7.6) |
| Congestive heart disease | 245 (29.6) | 55 (14.3) |
| Myocardial infarction | 210 (25.3) | 80 (20.8) |
| Cerebrovascular disease | 127 (15.3) | 39 (10.2) |
| Chronic pulmonary disease | 167 (20.1) | 36 (9.4) |
| Liver disease | 32 (3.9) | 12 (3.1) |
| Vascular disease | 244 (29.4) | 89 (23.2) |
| Tumor | 141 (17.0) | 22 (5.7) |
| Lymphoma | 12 (1.4) | 3 (0.8) |
| Leukemia | 8 (1.0) | 4 (1.0) |
| Metastasis | 28 (3.4) | 5 (1.3) |
| **Year of treatment, n (%)** |  |  |
| 2005-2006 | 139 (16.8) | 105 (27.3) |
| 2007-2008 | 155 (18.7) | 66 (17.2) |
| 2009-2010 | 162 (19.5) | 72 (18.8) |
| 2011-2012 | 185 (22.3) | 67 (17.4) |
| 2013-2014 | 188 (22.7) | 74 (19.3) |
| Abbreviations: ICU: intensive care unit, IQI: interquartile interval, N: number, RRT: Renal replacement therapy, SD: Standard deviation, SOFA: sequential organ assessment score. | | |
|
