# Supplementary material for: Timing of renal replacement therapy and long-term risk of chronic kidney disease and death in intensive care patients with acute kidney injury
Source: Crit Care. 2017 Dec 28;21:326. doi: 10.1186/s13054-017-1903-y (PMC5745999; doi:10.1186/s13054-017-1903-y)
Supplement: Supplementary file 5 — Sensitivity analyses. (DOC 41 kb) [file 13054_2017_1903_MOESM5_ESM.doc]

**Additional file 5: Table S5** Sensitivity analyses

|  | IPT-weighted analysis after exclusion of patients treated with ECMO | | |
| --- | --- | --- | --- |
|  | Early RRT, % | Late RRT, % | HR (95% CI) |
| Mortality, N = 1064 |  |  |  |
| 0 to 90 days | 55.2 | 45.2 | 1.31 (1.09-1.59) |
| 90 days to 5 years | 41.3 | 42.5 | 1.04 (0.76-1.42) |
| 0 to 5 years | 73.7 | 68.5 | NA |
| CKD, N = 268 |  |  |  |
| 90 days to 5 years | 35.8 | 45.9 | 0.72 (0.44-1.17) |
| ESRD, N = 536 |  |  |  |
| 90 days to 5 years | 15.5 | 18.3 | 0.84 (0.47-1.50) |
|  | IPT-weighted analysis after a 5th percentile asymmetrical trim | | |
|  | Early RRT, % | Late RRT, % | HR (95% CI) |
| Mortality, N = 900 |  |  |  |
| 0 to 90 days | 53.0 | 46.4 | 1.23 (1.01-1.49) |
| 90 days to 5 years | 35.7 | 39.9 | 0.96 (0.69-1.35) |
| 0 to 5 years | 69.8 | 67.8 | NA |
| CKD, N = 188 |  |  |  |
| 90 days to 5 years | 35.2 | 44.9 | 0.75 (0.46-1.21) |
| ESRD, N = 417 |  |  |  |
| 90 days to 5 years | 14.3 | 13.9 | 1.02 (0.59-1.75) |
| Abbreviations: CI: Confidence interval, CKD: Chronic kidney disease, ECMO: Extracorporeal membrane oxygenation, ESRD: End-stage renal disease, HR: Hazard ratio, IPT: Inverse probability of treatment, NA: Not applicable, RRT: Renal replacement therapy. | | | |
|
|
